# Solarplast^®^ Demonstrates Anti-Inflammatory and Anti-Oxidant Activity In Vivo and Positively Modulates Perceived Anti-Ageing Quality of Life Questionnaire and Skin Analogue Scale

**DOI:** 10.3390/ijms252312689

**Published:** 2024-11-26

**Authors:** Kieran Rea, Antonio M. Inarejos-Garcia, Sonia Guilera Bermell, Reme Garcia Bou, Yinka Olusoga, John Deaton

**Affiliations:** 1 ADM Cork H&W Limited, Food Science Building, University College Cork, T12 Y337 Cork, Ireland; 2 Department of Functional Extracts, ADM Valencia, 46740 Carcaixent, Spain; antonio.inarejos@adm.com (A.M.I.-G.); sonia.guilera@adm.com (S.G.B.); reme.garcia@adm.com (R.G.B.); 3 ADM Deerland Probiotics and Enzymes, 3800 Cobb International Boulevard, Kennesaw, GA 30152, USA; yinka.olusoga@adm.com (Y.O.); john.deaton@adm.com (J.D.)

**Keywords:** Solarplast, antioxidant, anti-inflammatory, anti-ageing, skin

## Abstract

Solarplast^®^ is an organic, non-GMO (genetically modified organism) dietary supplement from an enzymatically treated spinach preparation containing numerous active components that exhibit antioxidative and anti-inflammatory properties. The purpose of this study was to evaluate the effects of a 45-day supplementation period in adult men and women (Total *n* = 84), some of whom were classified as “everyday smokers”. The main outcomes include metabolic readouts, oxidative stress, inflammation, and secondary subjective assessments, including skin, physical, and mental health questionnaires. Solarplast^®^ attenuated some markers associated with smoking-induced increases in inflammatory tone and oxidative stress markers. Furthermore, Solarplast^®^ administration improved anti-ageing quality of life mental scores associated with depression-related symptoms, loss of self-confidence, and some anxiety-related symptoms and exhibited positive effects in some readouts of anti-ageing quality of life physical scores and skin visual analogue scores. In summary, Solarplast^®^ is safe, well-tolerated, may reduce circulating inflammatory and oxidative stress markers, and may positively impact some mental and physical quality-of-life parameters as well as skin quality.

## 1. Introduction

Oxidative stress can be defined as the disturbance in the balance between the production of reactive oxygen species (i.e., free radicals) and antioxidant defenses. Reactive oxygen species (ROS) and reactive nitrogen species (RNS) are produced continuously in the body via oxidative metabolism, mitochondrial bioenergetics, and immune function throughout the lifespan. Organisms, such as humans, experience an increased burden of oxidative stress for a host of reasons including environmental factors, such as toxins in the air and sunlight, food consumption (saturated fat and refined carbohydrates in particular) as well as other miscellaneous reasons including exercise, smoking, medication, and alcohol consumption.

Humans have a well-developed, complex defense system against oxidative stress; however, the effectiveness of said defense may decrease due to environmental factors and as we age [1,2,3,4]. In turn, there is an increase in the failure of biochemical mechanisms influencing physiological conditions such as fatigue, memory loss, muscle and/or joint pain, wrinkles, and grey hair, decreased eyesight, headaches, and susceptibility to infections, often dependent on the extent of the exposure. Inflammation is a manifestation of oxidative stress, and the pathways that generate the mediators of inflammation are all induced by oxidative stress [5,6]. Dietary consumption of foods or supplements with antioxidants may prove beneficial to health by alleviating the effects of oxidative stress and subsequent changes in inflammatory markers.

Green leafy vegetables naturally high in antioxidants are an important component of a balanced healthy diet, with low caloric value yet a rich source of nutrients, high in dietary fiber, low in lipids, and rich in foliate, vitamin C (ascorbic acid), vitamin K (phylloquinone), magnesium, and potassium. Mineral nutrients like iron and calcium are also high in leafy vegetables. Furthermore, leafy vegetables are the only natural sources of folic acid, which are considerably high in the leaves of spinach, asparagus, lettuce, mustard green, colocasia green leaf, and turnip green plants. Research suggests that supplementing the diet with green leafy vegetables could provide a source for antioxidants and the nutritional requirements necessary for proper growth as well as adequate protection against diseases caused by malnutrition [7]. Spinach or *Spinacia oleracea* contains numerous active components including flavonoids, carotenoids such as beta-carotene (pro-vitamin A), lutein, folate, vitamin C, calcium, iron, phosphorous, sodium, and potassium that exhibit antioxidative and anti-inflammatory properties [8,9]. Spinach also has one of the highest Oxygen Radical Absorbance Capacity (ORAC) values of any vegetable [10].

Solarplast^®^ is an enzymatically treated spinach preparation derived from *Spinacia oleracea* where organic, frozen spinach undergoes proprietary processing with an enzyme formulation under specific conditions to concentrate the active components and make them more bioavailable. The enzymatic processing is then stopped, and the resulting supplement is freeze-dried to produce a lyophilized spinach preparation. The mechanistic components of Solarplast^®^ consist of antioxidants normally found in the body, including glutathione enzymes, reductase, peroxidase, catalase, superoxide dismutase (SOD), nicotinamide adenine dinucleotide phosphate (NADPH), and flavin adenine dinucleotide (FAD). Additionally, Solarplast^®^ contains photosynthetic complexes with high concentrations of adenosine triphosphate (ATP), NADPH, adenosine diphosphate (ADP), adenosine monophosphate (AMP), NADP, niacin, vitamin B12, adenine, and ribose. Recently, Solarplast^®^ in vitro evidence demonstrated superior antioxidant activity when compared to frozen spinach leaves in total oxidant capacity (TOC), Ferric Reducing Antioxidant Power (FRAP), and trolox-equivalent antioxidant capacity (TEAC) antioxidant assays. Several antioxidant enzymes were also increased in Solarplast^®^, when compared to frozen spinach. As a functional readout, Solarplast^®^ attenuated hydrogen peroxide-, ethanol-, and acetaminophen-induced increases in oxidative stress and cytotoxicity in both intestinal (HT-29) and liver (HepG2) cell lines [11].

Nutritional supplementation to obtain antioxidants has garnered great interest over the last decades, as many people do not consume enough fruits and vegetables, which are our primary food sources of antioxidants. Herein, the antioxidant potential of Solarplast^®^ was characterized using a total phenolic compounds assessment, ORAC assessment, DPPH assessment (2,2-diphenyl-1-picrylhydrazyl), FRAP analysis, and flavonoid analysis. These are all commonly utilized methods in plant biochemistry to evaluate and characterize the properties of plant constituents for scavenging free radicals and antioxidant potential. An exploratory clinical study was then undertaken to evaluate the effects of a 45-day Solarplast^®^ supplementation period on readouts associated with oxidative stress and inflammatory markers in adult men and women, some of whom were smokers. Smokers were selected as a subgroup as it is well known that cigarette use enhances oxidative stress by mounting reactive oxygen species and weakening antioxidant defense systems [12].

From a safety perspective, there were no adverse events reported in this study, and there was no negative impact on any of the measured serum metabolites. In the non-smoking study participants, 45-day Solarplast^®^ supplementation had no negative impact on oxidative stress markers or inflammatory markers and reduced serum levels of the pro-inflammatory marker TNFα. In the smoking population, there was an overall increase in serum oxidative stress markers and inflammatory markers. The 45-day Solarplast^®^ supplementation attenuated the smoking-induced decrease in glutathione and attenuated the smoking-induced increase in ROS/RNS ratio, which is an index of oxidative health status. Furthermore, Solarplast^®^ attenuated the smoking induced increase in the inflammatory markers IL-6 and TNFα but not the smoking-induced increase in IL-4.

Using the anti-ageing quality of life questionnaire, the impact of the 45-day Solarplast^®^ supplementation on symptoms related to physical and mental readouts was assessed. There are no validated questionnaires to assess oxidative health status per se, but this questionnaire is used as a screening tool for healthy ageing, which can be influenced by oxidative health status. Our hypothesis was that if Solarplast^®^ supplementation can attenuate negative physiological effects of oxidative stress and inflammation, then this may be captured in a self-reported questionnaire on quality of life. The 45-day Solarplast^®^ supplementation improved anti-ageing quality of life mental scores associated with depression-related symptoms, loss of self-confidence, and some anxiety-related symptoms and exhibited positive effects in some readouts of anti-ageing quality of life physical scores.

The impact of 45-day Solarplast^®^ supplementation was further explored in a non-validated questionnaire related to skin health using a self-reported questionnaire with a visual analogue scale. Oxidative health status has been linked to skin quality [13], and the hypothesis was that if Solarplast^®^ could improve oxidative health status, it may improve skin quality in the study participants. The 45-day Solarplast^®^ supplementation had an overall improvement in two readouts of this questionnaire, improving elasticity of skin and a glossy appearance of skin, as determined by self-assessment by the study participants.

Overall, this exploratory study provides evidence that 45-day Solarplast^®^ supplementation may improve oxidative health status and inflammatory tone and improve symptoms related to oxidative health. Further clinical studies with longer administration period and a larger sample size are warranted.

## 2. Results

### 2.1. Solarplast^®^ Characterization

#### Antioxidant and Chromatographic Analyses

The antioxidant characterization results from various assays including FRAP (Ferric Reducing Antioxidant Power), free radical DPPH test, ORAC (Oxygen Radical Absorbance Capacity), and TPC (Total Polyphenols Content) demonstrate the anti-oxidant potential of Solarplast^®^ (Table 1).

**Figure 1:**
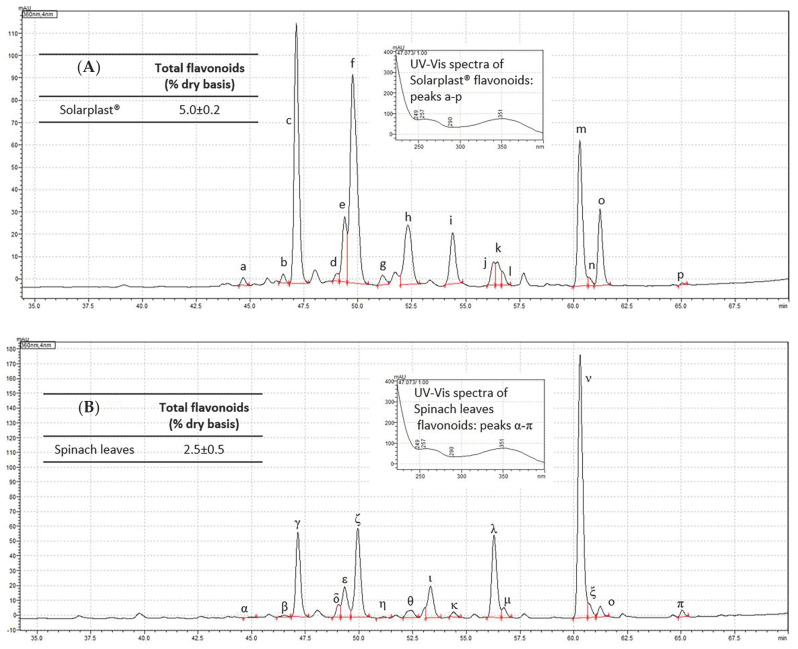
The identification of phenolic spectra and quantification of phenolic components in (**A**) Solarplast^®^ (labelled a–p) and (**B**) spinach leaves (Greek letters from “α” to “π”) was focused on the flavonoids, which were major components. At least 16 of the 22 separated peaks in each chromatogram (about 70%) showed characteristic UV-Vis spectra of flavonoids, mostly flavones, flavonols and the corresponding glycosides, usually detected at 254–280 nm or 340–360 nm. Solarplast^®^ reported a total flavonoid concentration by HPLC that was double that of spinach leaves (5.0 ± 0.2 vs. 2.5 ± 0.5%, dry basis).

The comparison of the phenolic components present in Solarplast^®^ and spinach leaves was performed by chromatographic analysis and showed a similar chromatographic profile (Figure 1), suggesting that the phenolic compounds of spinach are still present in Solarplast^®^ after the manufacturing process.

For the quantification of total flavonoids in the preparations, PDA detection was performed at 360 nm using an external calibration curve with at least five different calibration points (r > 0.99) of luteolin-7-O-glucoside for the quantification of total flavonoids with the same characteristic UV-Vis spectra (Figure 1). The sum of flavonoids was expressed as luteolin-7-O-glucoside equivalents (%, dry basis).

In a separate methodology using HPLC coupled to mass spectrometry, two phenolic acids and 17 flavonoids were identified in Solarplast^®^ compared with the MS data from the literature (Table 2). Using HPLC-DAD, it is not possible to discriminate all of the different phenolic compounds that may elute at the same retention time. The combination of HPLC with mass spectrometry confirms that some phenolic compounds elute at similar times, and there are some peaks in the chromatogram that remain unidentified except for the fact that they belong to the flavonoid family as determined by their UV-Vis spectra. At least four groups of flavonoids were differentiated: flavonols, glycoside flavonols, flavones, and one flavanone. Within flavonols, three different patuletin specieswere identified, showing three deprotonated molecules in negative mode at *m*/*z* 787.1952 and fragment ion at *m*/*z* 332.0499 (Patuletin-3-O-*β*-D-glucopyranosyl-(1→6)-β-D-apiofuranosyl-(1→2)]-β-D-glucopyranoside), *m*/*z* 934.2396 and fragment ion at *m*/*z* 331.0445 (Patuletin-3-O-β-D-(2′′-p-coumaroylglucopyranosyl-(1→6)-[β-D-apiofuranosyl-(1→2)]-β-D-glucopyranoside), and *m*/*z* 656.1593 and fragment ion at *m*/*z* 331.0555 (Patuletin-3-O-β-D-glucopyranosyl-(1→6)-β-D-glucopyranoside). The other flavonols were spinacetin, with deprotonated molecules at *m*/*z* 801.2115 and fragment ion at *m*/*z* 346.0633 (Spinacetin-3-O-β-D-glucopyranosyl-(1→6)-[β-D-apiofuranosyl-(1→2)]-β-Dglucopyranoside), *m*/*z* 670.1731 with the fragment ion at *m*/*z* 346.0642 (Spinacetin-3-O-β-D-glucopyranosyl-(1→6)-β-D-glucopyranoside), myricetin (*m*/*z* 317.0277), quercetin (*m*/*z* 301.0332), and kaempferol (*m*/*z* 285.2698) (Table 2). Two glycoside flavonols were identified as quercetin-3-O-grutinoside (*m*/*z* 609.0482) and quercetin-3-O-glucoside (*m*/*z* 463.2931), and seven flavones such as luteolin-7-O-glucoside (*m*/*z* 448.1897), apigenin-7-O-glucoside (*m*/*z* 431.2255), luteolin (*m*/*z* 285.2692), apigenin-7-O-rutinoside (*m*/*z* 577.3579), luteolin-7-O-rutinoside (*m*/*z* 593.3925 and at *m*/*z* 283.2637), and apigenin (*m*/*z* 269.2468). Finally, regarding flavanones, only naringenin was identified with the deprotonated molecule at *m*/*z* 271.2286 (Table 2).

### 2.2. Clinical Study

#### 2.2.1. Anthropometric and General Characteristics

Physical anthropometric characteristics did not differ between Solarplast^®^ and placebo groups (Table 3). There was no effect of treatment, smoking status, or interaction on height [F(1, 80) = 0.2831, *p* = 0.5961; F(1, 80) = 0.0757, *p* = 0.7839; F(1, 80) = 0.1485, *p* = 0.7010], body mass [F(1, 80) = 0.0158, *p* = 0.9003; F(1, 80) = 1.440, *p* = 0.2336; F(1, 80) = 0.6585, *p* = 0.4195], or body fat percentage [F(1, 80) = 0.3713, *p* = 0.5440; F(1, 80) = 0.4584, *p* = 0.5003; F(1, 80) = 2.219, *p* = 0.1403]. There were no adverse effects reported for either condition (i.e., placebo or Solarplast^®^), and all conditions appeared to be well tolerated.

In the non-smoking placebo group, 30 of the 35 individuals were below 30, and the range was from 20 to 57 years old. In the non-smoking Solarplast^®^ treatment group, 29 of the 33 participants were below 30, and the range was from 22 to 56 years old. In the smoking placebo group, five of the individuals were below 30, and the range was from 22 to 56 years old. In the smoking Solarplast^®^ treatment group, five of the individuals were below 30, with a range from 22 to 59 years old.

In the participants that were smokers, there was no significant difference between the average number of cigarettes smoked per day between the placebo and Solarplast^®^ groups (Table 3). The number of cigarettes ranged from 1 to 15 per day in the placebo group and 1 to 12 in the Solarplast^®^ group. In both groups, only one participant smoked more than 10 cigarettes per day.

All participants confirmed they were not on any specific dietary plan (ketogenic, vegan, vegetarian, fasting, intermittent fasting, Mediterranean) and did not alter their dietary pattern or physical exercise levels over the duration of this study.

#### 2.2.2. Comprehensive Metabolic Panel

There were no significant changes for glucose, BUN, creatinine, BUN to creatinine ratio, sodium, potassium, chloride, carbon dioxide, calcium, total protein, albumin, globulin, A/G ratio, bilirubin, alkaline phosphatase, AST, ALT, and EGFR (Table 4).

#### 2.2.3. Oxidative Stress

**Figure 2:**
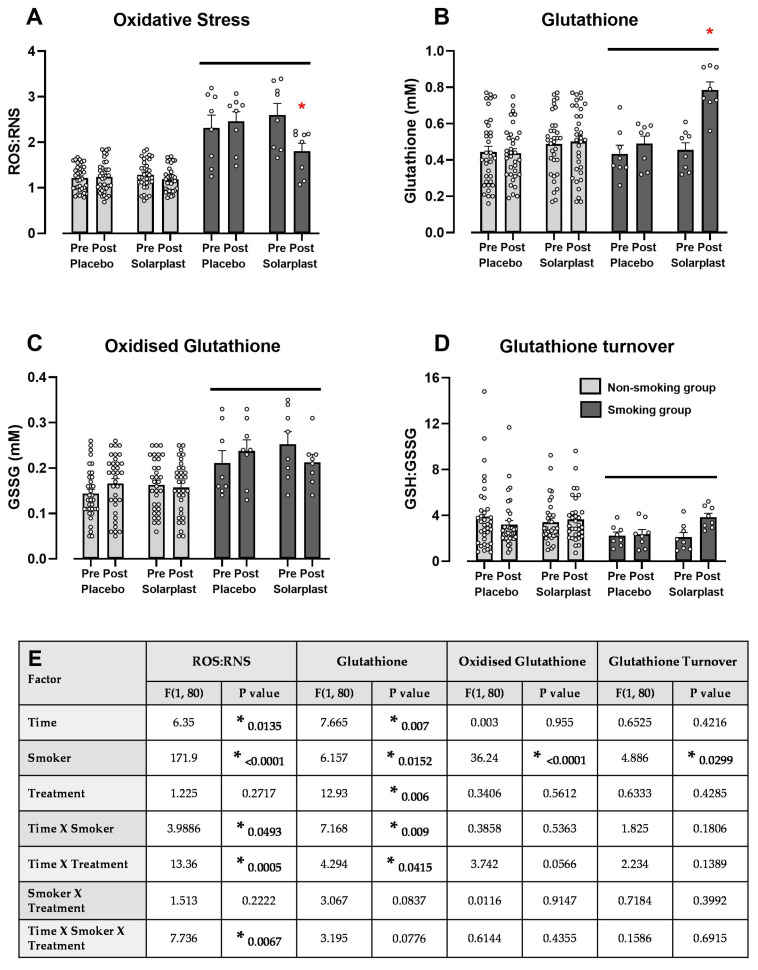
Solarplast^®^ significantly attenuated smoking-induced changes in antioxidant biomarkers following 45 days of supplementation (**A**–**D**), with the three-way ANOVA results below (**E**). Solid line signifies a significant overall effect of the smoking group. * Represents a significant effect (*p* < 0.05) of Solarplast^®^ at the end of the treatment as compared with the start of the treatment in the smoking group. All data are mean ± SEM.

There was an overall effect of smoking on oxidative stress markers observed as compared to the non-smoking group, as indicated by a solid bar (Figure 2A–D). Measuring the total free radical presence in a sample with reactive oxygen species and reactive nitrogen species (ROS/RNS) as an index, Solarplast^®^ significantly attenuated smoking-induced increases in oxidative stress (Figure 2A). Solarplast^®^ also significantly increased glutathione levels in the smoking group (Figure 2B), while having no further post-hoc significant effect on smoking-induced increases in oxidized glutathione (Figure 2C) or smoking-induced decreases in glutathione turnover (Figure 2D) between the respective groups. The statistical results of the three-way ANOVA are presented in Figure 2E.

#### 2.2.4. Inflammatory Tone

**Figure 3:**
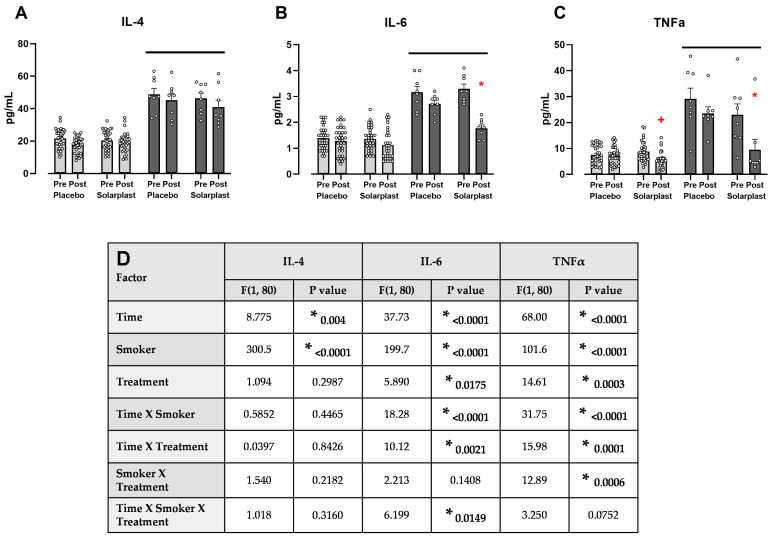
Solarplast^®^ significantly attenuated smoking-induced increases in (**B**) IL-6 and (**C**) TNFα but not for (**A**) IL-4 following 45 days of supplementation, with the three-way ANOVA results below (**D**). Solid line signifies a significant overall effect of the smoking group. * represents the significant post-hoc effect (*p* < 0.05) of Solarplast^®^ at the end of the treatment as compared with the start of the treatment in the smoking group. + represents a significant post-hoc effect (*p* < 0.05) as compared with baseline control in the Solarplast^®^ non-smoking group. All data are mean ± SEM.

There was an overall effect of smoking on inflammatory tone as compared to the non-smoking groups, as indicated by a solid bar (Figure 3A–C). Solarplast^®^ significantly attenuated smoking-induced increases in IL-6 (Figure 3B) and TNFα (Figure 3C). Furthermore, Solarplast^®^ significantly reduced TNFα in the non-smoking group (Figure 3C). The statistical analyses for the three-way ANOVA are presented in Figure 3D.

#### 2.2.5. Skin Questionnaire

**Figure 4:**
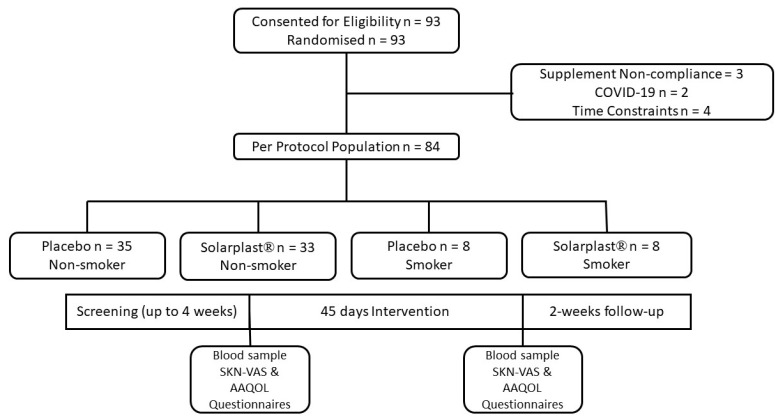
CONSORT flow diagram to illustrate the progress of the study participants.

There was no significant effect of smoking, treatment, or interaction for the change from baseline of various readouts in the skin questionnaire including dryness, flushing, inconsistency of make-up, itching of skin, eczema, wrinkles on body or face, course skin, skin softness, and overall complexion (Figure 4). However, Solarplast^®^ treatment improved elasticity of skin [F(1, 80) = 5.310, *p* = 0.0238] and glossy skin [F(1, 80) = 4.379, *p* = 0.0395], while there was no effect of smoking and no interaction effect as compared to the placebo control. There was no post-hoc difference between the respective groups (Table 5).

#### 2.2.6. Anti-Aging QOL Common Questionnaire: Physical Symptoms

Readouts from the anti-aging quality of life common questionnaire physical are captured under the headings of VDT-related symptoms, fatigue-related symptoms, persistent neurological symptoms, autonomic nerve-related symptoms, and fragile constitution. Only statistical analyses where we determined an effect of treatment, smoking, or interaction are reported.

- VDT-Related Symptoms

From the symptoms captured under VDT-related symptoms (tired eyes, blurry eyes, eye pain, stiff shoulders, muscle pain/strain, lethargy, headache), Solarplast^®^ treatment significantly reduced tired eyes [F(1, 80) = 5.027, *p* = 0.0277], while there was no effect of smoking and no interaction effect. Solarplast^®^ treatment also significantly reduced the sensation of blurry eyes where there was a treatment effect [F(1, 80) = 5.810, *p* = 0.0182] and a smoking effect [F(1, 80) = 4.544, *p* = 0.0361] but no interaction effect. There was no significant difference between individual groups as determined by post-hoc comparisons. Still shoulders were perceived to be less in the smoking group [F(1, 80) = 5.552, *p* = 0.0209] as compared with the non-smoking group, but there was no effect of Solarplast^®^ or any interaction effect and no post-hoc difference between groups (Table 6).

- Fatigue-Related Symptoms

Of the fatigue-related symptoms (overweight, lethargy, no feeling of good health, appetite loss, early satiety, epigastralgia), there was no smoking-related effect, treatment effect, or interaction effect on any readouts (Table 6).

- Persistent Neurological Symptoms

There were no changes in persistent neurological symptoms (palpitation, thirst, headache, dizziness, tinnitus, lumbago, arthralgia) following Solarplast^®^ administration. However, there was an overall reduction in perceived thirst in the smoking population [F(1, 80) = 6.150, *p* = 0.0152], but there was no effect of smoking or interaction effect and no post-hoc significance between groups (Table 6).

- Autonomic Nerve-Related Symptoms

Of the autonomic nerve-related symptoms (dizziness, tinnitus, edema, sweating, frequent urination, hot flush, cold sensation), Solarplast^®^ attenuated the perceived incidence of sweating [F(1, 80) = 4.201, *p* = 0.0437] after 45 days of treatment, while there was no effect of smoking or an interaction effect. However, there were no post-hoc significant differences between any groups (Table 6).

- Fragile Constitution

For fragile constitutions, weight loss, skin problems, weak chest, coughing and sputum, diarrhea, constipation, and cold sensations were assessed. Solarplast^®^ attenuated the perceived incidence of skin problems [F(1, 80) = 4.816, *p* = 0.0311], while there was no effect of smoking nor an interaction effect. There was a significant effect of smoking [F(1, 80) = 8.133, *p* = 0.0055] and treatment [F(1, 80) = 6.408, *p* = 0.0133], for perceived cough and sputum, and there was also an interaction effect [F(1, 80) = 4.395, *p* = 0.0392] (Table 6).

#### 2.2.7. Anti-Aging QOL Common Questionnaire: Mental Symptoms

Readouts from the anti-aging quality of life common questionnaire for mental symptoms are captured under the headings of depression-related symptoms, loss of self-confidence, and anxiety-related symptoms. Only statistical analyses where we determined an effect of treatment or smoking or an interaction effect are reported.

- Depression-Related Symptoms

Solarplast^®^ had a strong effect on perceived depression-related symptoms following 45 days treatment, reducing irritability [F(1, 80) = 4.296, *p* = 0.0414], easily angered [F(1, 80) = 12.96, *p* = 0.005], reluctance to talk with others [F(1, 80) = 4.318, *p* = 0.0409], and feeling depressed [F(1, 80) = 20.15, *p* < 0.0001]. There was an overall decrease in feelings of uselessness in the smoking group [F(1, 80) = 4.162, *p* = 0.0446] but no post-hoc difference between groups (Table 7).

- Loss of Self-Confidence

Solarplast^®^ had a strong effect on loss of self-confidence following 45 days of treatment reducing loss of confidence [F(1, 80) = 4.620, *p* = 0.0346], loss of motivation [F(1, 80) = 3.973, *p* < 0.0496], and perceived lack of something to look forward to ([F(1, 80) = 10.80, *p* = 0.0015], with a smoking X treatment interaction effect [F(1, 80) = 10.92, *p* < 0.0014]), and also attenuated smoking-induced increase in inability to sleep because of worries [F(1, 80) = 12.45, *p* = 0.0007] (Table 7).

- Anxiety-Related Symptoms

Solarplast^®^ reduced the perceived sense of tension [F(1, 80) = 5.487, *p* = 0.0216] and feeling anxious for no apparent reason [F(1, 80) = 4.423, *p* = 0.0386] following 45 days of treatment. However, there were no post-hoc significant differences between groups (Table 7).

### 2.3. Limitations

The present study is not without limitations. Firstly, while we conducted complete dietary food logs and tracking, we did not correct for individual dietary consumption and their intrinsic antioxidant activity (i.e., we did not restrict participants to specific diets). While participants were instructed not to change their exercise habits nor to engage in exercise 48 h prior to visiting the laboratory, we have no way of being completely sure participants followed these instructions or maintained a consistent level of physical activity for the duration of this study. However, all participants confirmed they were not on any specific dietary plan (ketogenic, vegan, vegetarian, fasting, intermittent fasting, Mediterranean) and did not alter their dietary pattern or physical exercise levels over the duration of this study. While the study design was to recruit individuals across an age range of 18–65, the majority of the participants in all groups were between 22 and 30 years of age. This may have reduced the likelihood of detecting a positive effect in the treatment groups, as in general, the efficacy of the body in scavenging free radicals and combating inflammation decreases as we age. A related observation would be that in the smoking groups, many individuals would have been smoking for a relatively short duration of time as compared to older individuals in the smoking groups. Our sample size for our smoking group was small, and other antioxidant markers such as low-molecular-mass vitamins A, C and E, uric acid, tocopherol, β-carotene, β-cryptoxanthin, lutein, zeaxanthin, lycopene, superoxide dismutase, catalase, TBARS, and retinol were not included in the analyses.

Nevertheless, despite the limitations of this study, we still detected a positive effect of Solarplast^®^ for several physiological readouts and in self-reported questionnaires. We feel that these preliminary findings in this exploratory study warrant further research. Future research should consider crossover design, with more analytes being investigated and longer supplementation, although we do see physiologically relevant improvements in biometrics and a self-reported questionnaire with this study design and timeframe. Further, the investigation in clinical populations that experience high levels of oxidative stress and inflammation and/or older cohorts who may benefit from the supplement may be interesting to explore.

## 3. Discussion

The human diet represents a source of many different compounds endowed with antioxidant activity, mainly vitamins, polyphenols, and flavonoids. While the intake of fruits and vegetables worldwide remains below the recommended daily amount, the beneficial effects on health of a diet rich in fruits and vegetables are well known in the general population [14]. Additionally, once harvested, and throughout distribution and storage, the antioxidant activity of fruits and vegetables is reduced through a plethora of reduction–oxidation reactions [15]. Together these represent a worrying trend, and thus, for many people, supplementation is warranted as the routine consumption of antioxidants is proposed to reduce the risk of lifestyle-related illnesses such as diabetes, obesity, and cardiovascular disease [16].

According to Gil and colleagues [17], the storage process postharvest reduced the content of antioxidants such as vitamin C and the scavenging activity of flavonoids in spinach. Solarplast^®^ is unique in that through a proprietary process, organic spinach is enzymatically enhanced and capitalizes on protoplast (isolated plant cells that lack the rigid cellulose walls found in intact tissue) activity. To date, a number of studies have demonstrated the antioxidant activities of spinach in various forms (including powdered and raw) [17,18,19], and we have also recently demonstrated the superior antioxidant efficacy of Solarplast^®^ as compared with spinach preparation alone in vitro [11]. In this manuscript, we provide further evidence for the antioxidant potential of Solarplast^®^ with different spectrophotometric methodologies (FRAP, DPPH, ORAC, and Total Polyphenols). The higher antioxidant activity and dry storage represent a possible, stable supplementation to help address the need for additional antioxidants.

Our exposure to environmental factors, such as cigarette smoking [20] and other lifestyle factors may also contribute to the plethora of symptoms and disorders related to oxidative stress. In one puff of a cigarette, it is estimated that there are greater than 1 × 10^15^ free radicals that contribute to oxidative stress and damage to the lung and other tissues in vivo [21]. Clinical studies, though predominantly epidemiological studies, have demonstrated that systemic oxidative stress observed in cigarette smokers can occur as a result of direct exposure to oxidizing agents contained in cigarette smoke as well as indirectly through the activation of inflammatory responses resulting from exposure to cigarette smoke constituents [22,23,24]. It is generally accepted that the large number of chemical agents in cigarette smoking, including reactive oxygen and nitrogen species (ROS and RNS), saturated aldehydes (e.g., acetaldehyde), and reactive α,β-unsaturated aldehydes (e.g., acrolein, 4-hydroxy-2-nonenal, and crotonaldehyde) [25] induce adverse effects on cells, tissues and organs through oxidative damage to key biological molecules [26,27]. The oxidative burden detected in cigarette smokers can occur either via direct oxidative damage to biomolecules and/or via indirect pathways, such as oxidants-antioxidants imbalance, lipid peroxidation, or protein carbonylation [27]. In a recent review, evidence for the lowering of almost all low-molecular-mass antioxidants, including glutathione [28] was presented as compared to non-smoking controls [27].

Cigarette smoking also stimulates an inflammatory response [20] including TNFα [29], IL-4 [30] and IL-6 [31,32]; characterized by the activation of macrophages and the recruitment/activation of neutrophils, eosinophils, monocytes and lymphocytes possibly through the activation of the nuclear factor (NF)-κB pathway via the generation of ROS/RNS and aldehydes, such as acrolein and crotonaldehyde [33]. As such, this demographic was assessed in parallel with non-smoking individuals for antioxidant and anti-inflammatory efficacy and readouts in skin, and anti-ageing questionnaires.

In the non-smoking group, Solarplast^®^ significantly decreased TNFα levels as compared to baseline levels in the non-smoking groups, demonstrating a positive effect on daily environmental impact on inflammatory tone in the absence of any obvious environmental stressor (e.g., smoking). In the smoking demographic, there was a significantly increased level of IL-6, IL-4 and TNFα which support earlier findings from the literature. Solarplast^®^ supplementation attenuated smoking-induced increase in inflammatory tone, specifically in IL-6 and TNFα concentrations as well as raising glutathione levels.

In the smoking groups, as compared to the non-smoking groups there was also an altered overall oxidative stress status with an increased ROS/RNS ratio, elevated oxidized glutathione, and decreased glutathione turnover. This effect of smoking on ROS/RNS ratio was attenuated following 45 days of Solarplast^®^ supplementation. Solarplast^®^ further increased glutathione levels in the smoking demographic, suggesting it may also support antioxidant activity in this demographic.

Interleukin-6 (IL-6) is a multifunctional cytokine that participates in inflammatory and immune responses. Its immunological activities include B cell differentiation and stimulation of IgG secretion, T cell differentiation and growth, and cytotoxic T cell differentiation [34]. IL-6 is produced by activated monocytes, macrophages, endothelial cells, fibroblasts, keratinocytes, and activated T and B cells in response to induction by various stimuli, including other cytokines [35]. The functions of TNF-α are mediated through its two main receptors: tumor necrosis factor receptor 1 (TNFR1) and tumor necrosis factor receptor 2 (TNFR2) [36,37]. The activation of TNFR1 is known to initiate inflammatory, apoptotic, and degenerative cascades. In contrast, TNF-α signaling through TNFR2 is anti-inflammatory and cytoprotective, resulting in the induction of proliferation, differentiation, angiogenesis, and tissue repair. TNF-α can induce the secretion of IL-6 by keratinocytes, macrophages, and endothelial cells [36,37]. Interleukin 4 is a potent regulator of immunity secreted primarily by mast cells, Th2 cells, eosinophils, and basophils may be associated with increased serum levels of IgE, which is commonly associated with cigarette smoking [30]. Our observations suggest a smoking-induced increase in inflammatory serum cytokines, IL-4, IL-6, and TNFα, that was attenuated with dietary Solarplast^®^ supplementation. This effect may be due to some antioxidant activity of Solarplast^®^, thus suppressing the inflammatory response driven by oxidative stress markers. Glutathione (GSH) is a non-enzymatic antioxidant, which detoxifies free radicals or the byproducts of their reactions either directly or indirectly through reactions catalyzed by glutathione peroxidases and glutathione-S-transferase enzymes [38]. GSH also enhances the activity of other antioxidants, such as vitamin C and E, thereby elevating the overall antioxidant defense capacity [39]. However, the GSH antioxidant properties can be diminished or overwhelmed by excessive ROS generated during increased oxidative stress states such as cigarette smoking [40]. Reactive oxygen species (ROS) and reactive nitrogen species (RNS) are well-established molecules responsible for the deleterious effects of oxidative stress, and the ROS/RNS assay determines the total free radical presence in a given sample. In our non-smoking group, we saw no increase in glutathione or changes in the redox state due to a normal amount of oxidative stress, and Solarplast^®^ had no further effects on any readouts in this demographic. However, in the smoking group that received Solarplast^®^, there was an overall increase in glutathione and a non-significant decrease in oxidized glutathione (and a subsequent increase in the GSH/GSSG ratio) as compared with their baseline levels in the smoking Solarplast^®^ group. These findings suggest that Solarplast^®^ may aid in glutathione reduction and recycling during increased oxidative stress. However, further work investigating other oxidative stress markers is warranted to elucidate the potential mechanisms of action for this dietary supplement in influencing inflammatory cytokines and oxidative stress markers.

Skin, physical, and mental health were also assessed via self-reported questionnaires as part of this exploratory study to determine whether this anti-oxidant containing dietary supplement could positively modulate these readouts or attenuate any smoking-induced deficits. Using a self-reported visual analogue scale, participants reported on a number of skin readouts. It was determined that there was an overall improvement on elasticity of skin and glossy skin in the smoking and non-smoking groups in the treatment (Solarplast^®^) group as compared to placebo controls, but there were no post-hoc significant differences between different groups. There was no effect of smoking per se reported in any readout between smoking and non-smoking control groups. Among enzymatic antioxidants, glutathione peroxidase (GPx), catalase (CAT), and superoxide dismutase (SOD) are positively linked with skin health [41]. As such, Solarplast^®^ may have positively contributed to the cellular redox status of their skin; however, more research is needed on the exact mechanism.

Using the validated anti-ageing quality of life questionnaire, which captures perceived physical and mental symptoms, we saw a number of interesting findings. The only overall effects of smoking per se were in a reduction of the self-reported perception of stiff shoulders, a reduction in thirst, and a reduction in feelings of uselessness. However, there were no post-hoc differences between groups. Solarplast^®^ did not reverse any of these smoking-related self-reported readouts. These effects are difficult to interpret but may be driven by some of the psychoactive components in cigarettes. From the physical symptoms, there was an overall improvement of tired eyes, blurry eyes, and sweating in the Solarplast^®^-treated (smoking and non-smoking) groups as compared with placebo control groups, but there was no post-hoc significance between individual groups. There is no clear link between anti-oxidants and these parasympathetic physiological responses, but these observations may be due to some secondary effect of antioxidants on these readouts. In the Solarplast^®^-treated groups (smoking and non-smoking), there was an overall improvement in skin problems, with a significant perceived benefit in the non-smoking group as compared to non-smoking placebo controls as determined by post-hoc comparisons. These observations align with the skin questionnaire, suggesting a potential benefit for Solarplast^®^ in skin readouts. A reduction in coughing and sputum was also observed in the Solarplast^®^-treated groups as compared with placebo controls, largely being driven by the improvement in the smoking Solarplast^®^-treated group. This finding may be accounted for by an attenuation of smoking-induced phlegm production as a general side-effect of smoking; however, this parameter was not specifically recorded in this study.

Regarding the mental symptoms, Solarplast^®^ improved perceived scores in 10 out of 21 readouts across depression-related symptoms, loss of self-confidence readouts, and anxiety-related symptoms. Specifically, irritability, reluctance to speak with others, easily angered, feeling depressed, loss of motivation, nothing to look forward to, inability to sleep because of worries, sense of tension, and feeling anxious for no specific reason were all improved in the groups (smoking and non-smoking) receiving Solarplast^®^ supplementation, as compared to their placebo controls. Post-hoc analyses determined a significant difference between Solarplast^®^ and the placebo in the smoking groups for easily angered and for the smoking Solarplast^®^ group as compared with the non-smoking Solarplast^®^ group for loss of motivation and nothing to look forward to. Finally, there was a significant improvement in the feeling depressed readout where the Solarplast^®^ smoking group was significantly different from both the non-smoking Solarplast^®^ group and the smoking placebo group as determined by post-hoc comparisons. The potential mechanism behind how this dietary supplement rich in anti-oxidants can improve 10 out of the 21 self-reported questionnaires on mood and mental symptoms is difficult to determine without further evaluation but may be due to a decreased oxidative state and inflammatory tone contributing to the overall health in individuals leading to better moods. However, further studies are warranted to delineate these effects on mental readouts. These self-reported questionnaires suggest that following 45 days of supplementation with Solarplast^®^, there were moderate improvements in mood, skin, and some physical readouts that warrant further investigation.

## 4. Materials and Methods

Water, ethanol, and methanol of chromatographic and analytical grade together with acetic acid, sodium acetate (C_2_H_3_NaO_2_), hydrogen chloride (HCl), and sodium hydroxide (NaOH) of reagent grade were purchased from VWR (Valencia, Spain). DPPH (2,2-diphenil-1-1picrilhidracyl), Trolox (±)-6-Hydroxy-2,5,7,8-tetramethylchromane-2-carboxylic acid (97% purity), potassium persulphate (K_2_S_2_O_8_), ABTS [2,2′-azinobis-(3-ethylbenzothiazoline-6-sulfonic acid)], AAPH (azo initiator 2,2′-azobis(2-methyl-propanimidamide) dihydrochloride), TPTZ (2,4,6-tripyridyl-s-triazine), iron chloride hexahydrate (FeCl_3_·6H_2_O), iron sulphate heptahydrate (FeSO_4_·7H_2_O) as a standard reference, sodium phosphate dibasic (NaH_2_PO_4_), sodium phosphate monobasic (Na_2_HPO_4_), Fluorescein (FL), AAPH (2,2-azobis(2-amidino-propane) dihydrochloride), Folin-Ciocalteu reagent, anhydrous sodium carbonate, gallic acid (98% purity), and luteolin-7-O-glucoside (98% purity) were purchased from Merck (Madrid, Spain).

Solarplast^®^ was supplied by ADM^®^ Deerland Probiotic & Enzymes, Kennesaw, GA, USA. Solarplast^®^ was prepared from non-GMO, blanched frozen spinach using enzymatic digestion to remove the spinach cell walls, followed by freeze drying to produce a lyophilized spinach protoplast preparation.

### 4.1. Solarplast^®^ Characterization

#### 4.1.1. Total Polyphenols Content

Total Polyphenols Content (TPC) of Solarplast^®^ was carried out by means of the spectrophotometric method described by the International Organization for Standardization, ISO 14502-1:2005 [42], employing a nano-spectrophotometer (SPECTROstarNano BMG LABTECH) and expressing the Total Polyphenols concentration as gallic acid equivalents (%, dry basis).

#### 4.1.2. ORAC Assay

The antioxidant activity of Solarplast^®^ evaluated by the ORAC (Oxygen Radical Absorbance Capacity) test was analyzed according to [43]. AAPH was used as a peroxyl radical generator, Trolox as the standard, and fluorescein (sodium salt) as a fluorescent probe. The trials were performed on a Fluorimeter BioTek Synergy HTX Multimode Reader (Agilent Technologies, Barcelona, Spain), with excitation and emission wavelength of 485 and 535 nm, respectively.

#### 4.1.3. DPPH Assay

A free radical DPPH test was employed to evaluate the radical scavenging activity of Solarplast^®^ according to [44,45]. Briefly, the samples were dissolved in methanol and homogenized and finally filtered through a Nylon syringe filter (0.45 µm) before analyses by spectrophotometry (SPECTROstarNano BMG LABTECH, Biogen, Madrid, Spain) in 96 well-plates. Radical DPPH stock and working solutions (0.12 mM) were prepared in methanol together with a Trolox calibration curve (0.02–0.5 mM) for quantification of antioxidant activity at 517 nm.

#### 4.1.4. FRAP Assay

A Ferric Reducing Antioxidant Power (FRAP) assay was performed according to [46]. Briefly, the FRAP reagent was prepared by mixing 300 mM buffer acetate with 10 mM TPTZ and 20 mM FeCl_3_·6H_2_O, keeping the mixture at 37 °C for the formation of the iron complex Fe^3+^-TPTZ, (daily prepared). Samples diluted in ethanol (50%, *v*/*v*) were mixed with Fe^3+^-TPTZ for the corresponding reaction under darkness at 37 °C. The analyses were performed by spectrophotometry at a wavelength of 593–595 nm (SPECTROstarNano BMG LABTECH, Biogen, Madrid, Spain) until the stationary state. Trolox was used as reference standard for quantification (mM Trolox/g, dry basis).

#### 4.1.5. HPLC-DAD

The chromatographic analysis of flavonoids in Solarplast^®^ was performed by means of HPLC (High Performance Liquid Chromatography) coupled to a Diode Array Detector according to [47]. Briefly, Solarplast^®^ samples were solved in methanol (10 mg/mL) and homogenized and filtered through a Nylon syringe filter of 0.45 microns previous to injection. HPLC analyses were performed on a Shimadzu Nexera XR UHPLC 70 MPa with a Shimadzu SIL-40C XR autosampler and coupled to a photodiode array detector SPD-M40 model (Shimadzu, Madrid, Spain). The separation of flavonoids was performed on an octadecyl silane column Zorbax Eclipse Plus C18 (length 250 mm, ID 4.6 mm, particles 5 µm) protected by a corresponding precolumn (Agilent Technologies, Barcelona, Spain). The temperature of the column oven was set at 30 °C, the flow rate at 1.0 mL/min, and the injection volume at 20 µL. The gradient consisted of (A) water, 0.1% formic acid (*v*/*v*), and (B) methanol, starting at 5% B, increased to 15% B in 15 min, 30% B in 35 min, 40% B in 40 min, 50% B in 50 min, 60% B in 55 min, 75% B in 60 min, and 95% B in 65 min, and returned to the initial conditions 2 min later, with a maximum run time analysis of 70 min. For quantification, PDA detection was performed at 360 nm using an external calibration curve with at least five different calibration points (r > 0.99) of luteolin-7-O-glucoside for the quantification of total flavonoids with the same characteristic UV-Vis spectra. The sum of flavonoids was expressed as luteolin-7-O-glucoside equivalents (%, dry basis).

#### 4.1.6. HPLC-MS

For the identification of the individual phenolic components (phenolic acids and flavonoids) in Solarplast^®^, the same chromatographic conditions were used for the separation of the bioactive components [47], and the working flow was reduced to 0.5 mL/min for better ionization. Mass experiments were performed on a Q-TOF Impact II (Bruker, Hamburg, Germany) high-resolution mass spectrometer equipped with an electrospray ionization source. The source was operated in the negative ionization mode adjusted to 4000 V with an end plate offset potential of −500 V. The drying gas parameters were adjusted to 9.0 L min/min at 200 °C and gas pressure of mist at 3 bar, and data were collected in the range of *m*/*z* 60 to 1500.

### 4.2. Study Overview

This exploratory double-blind, parallel, placebo-controlled randomized study was prospectively registered as a Clinical Trial (NCT04144777) and was approved by the Kennesaw State University’s institutional review board (IRB #19-165). Participation consisted of two visits to the laboratory, separated by a 45-day supplementation period (Solarplast^®^ or placebo). During the supplementation period, participants completed two phone call check-ins with a research team member and logged their physical activity and dietary intake throughout their enrollment. The following primary outcome variables were specified a priori: blood biomarkers (complete metabolic panel, inflammation [TNF-α, IL-4, IL-6]), oxidative stress [glutathione, reactive oxygen species to reactive nitrogen species (ROS/RNS)] and perception of skin, physical, mental, and lifestyle outcomes via questionnaires. All data were collected in accordance with the Declaration of Helsinki.

### 4.3. Participants and Supplementation

Participants were recruited if they were (1) between the ages of 18–55 years, (2) biologically male or female, (3) smokers or non-smokers, (4) not trying to lose or gain weight, (5) not following a specific type of diet (e.g., ketogenic, vegetarian, fasting, etc.), (6) not consuming antibiotics or anti-inflammatory medications, (7) not an athlete, (8) willing to maintain their normal levels of physical activity and dietary patterns, and (9) free from non-communicable chronic diseases, including cardiovascular diseases, respiratory diseases, and diabetes. Smoking was defined as individuals who had smoked every day for the last year at the time of enrollment and termed “every day smoker”. Further, participants were excluded if they missed five or more days of the supplement, if they became pregnant, if they stopped smoking (if originally enrolled in the smoking group), if they started to take any new medications, and if they started a new physical activity or exercise routine. A total of 68 non-smoking individuals and 16 smokers completed the study of the 74 non-smokers and 19 smokers who were enrolled (drop-out/exclusion rate = 9%). The six non-smokers and three smokers that did not complete the study were excluded for the following reasons: (1) supplement non-compliance (two non-smokers, one smoker), (2) threat of COVID-19 (two non-smokers), and (3) time constraints associated with academic, family, or work responsibilities (two non-smokers, two smokers). Participants were divided into non-smokers and smokers and randomly assigned to consume either Solarplast^®^ (100 mg/day; 1 × 10^6^ light converting units [LCUs]) or the placebo (100 mg/day maltodextrin) once daily. The supplements were identical in texture, size, color, shape, and taste.

### 4.4. Test Visit Procedures

Participants arrived at the laboratory in a fasted state (8–12 h) for both visit 1 (Pre) and visit 2 (Post). Sessions took place in the morning between the hours of 8:00 a.m. and 12:00 EST. All visits for a given participant fell at approximately the same time (±2 h). Upon arrival for Pre, participants were informed of the study procedures, risks, and benefits and gave their verbal and written consent. They then filled out a health history questionnaire consisting of medical, physical activity, diet, and supplementation history and a 24 h food recall. Immediately following, participants had their anthropometrics and body composition assessed followed by a blood draw. The visit ended with completing questionnaires: skin visual analog scale (SKN-VAS) and Anti-Aging QOL Common Questionnaire (AAQOL). Prior to leaving, participants were given their randomly assigned supplement and instructions on how to take it each day for 45 days. The experimental capsules contained Solarplast^®^ (Deerland Probiotics & Enzymes; Kennesaw, GA, USA) comprised of a spinach preparation powder delivered with the carrier of rice dextrin and medium chain triglycerides at a daily dosage of 100 mg, while the placebo capsules consisted of rice dextrin and medium chain triglycerides. Participants then returned to the laboratory 45 days later for Post testing, which consisted of a supplement compliance assessment and the same tests and questionnaires as PRE (Figure 4).

### 4.5. Phone Call Check-Ins

Phone calls were completed by a research team member on days 15 and 30 of supplementation. These were intended to check on supplementation compliance (i.e., missed days taking the supplement) and check for adverse symptoms: skin—flushing/redness/warmth, itching, rash (appearance and location); throat symptoms; cough; back pain; abdominal pain; nausea/vomiting/diarrhea; fever; joint (pain/swelling/redness/stiffness); numbness/tingling; changes in blood pressure; dizziness; tunnel vision; loss of consciousness; confusion.

### 4.6. Anthropometrics and Body Composition

Body mass and height were assessed via a standard scale (WB-3000 Digital Scale, Tanita, Tokyo, Japan) without shoes. Body fat percentage was collected via bioelectrical impedance analysis (BIA; InBody770, InBody Co., Seoul, Republic of Korea) according to the manufacturer’s instructions.

### 4.7. Blood Collection and Analysis

Single venipunctures in the antecubital space were used to collect blood samples from participants. Approximately 20–25 mL of blood was taken from participants at each collection time. Samples were collected into Vacutainer™ tubes containing ethylenediaminetetraacetic acid (EDTA) and serum separator (SST) that were inverted 8–10 times prior to processing. SST tubes were set aside to clot for 10 min prior to centrifugation. Two separate centrifugation processes occurred. A single SST tube was centrifuged at room temperature (LabCorp Centrifuge Horizon Model 642E, Drucker Diagnostics, Port Matila, PA, USA) and picked up by a LabCorp representative for comprehensive metabolic panel assessment. The metabolic panel consisted of the assessment of glucose, blood urea nitrogen (BUN), creatinine, estimated glomerular filtration rate (EGFR), blood urea nitrogen–creatinine ratio, sodium, potassium, chloride, total carbon dioxide, calcium, total protein, albumin (A), total globulin (G), A/G ratio, total bilirubin, alkaline phosphatase, aspartate aminotransferase (AST), and alanine aminotransferase (ALT). The remaining SST and EDTA tubes were centrifuged at 1650× *g* for 10 min at 4 °C. The resulting serum and plasma were aliquoted and stored in a −80 °C freezer until analysis. Samples were aliquoted into multiple vials so as to not repeat freeze-thaw cycles. Samples were used to analyze markers of oxidative stress and inflammation per manufacturer guidelines and using a Varioskan LUX Multimode Microplate Reader (Thermofisher, Waltham, MA, USA): ROS/RNS (OxiSelect In Vitro ROS/RNS Assay Kit, STA-347-5, plasma) (Cell Biolabs, San Diego, CA, USA), glutathione (Cayman Chemical, 703002, plasma) (Cayman Chemical Company, Ann Arbor, MI, USA), IL-6 (Invitrogen, BMS213HS; serum) (Invitrogen, Waltham, MA, USA), IL-4 (Invitrogen, KHC0041; serum), and TNF-α (Invitrogen, BMS223HS; serum).

### 4.8. Questionnaires

The SKN-VAS was used to assess participants’ perceptions of skin changes and consisted of the following on a five-point scale: (1) dryness of skin; (2) flushing of the skin; (3) inconsistency of skin with make-up (if applicable); (4) itching of skin; (5) eczema; (6) body wrinkles; (7) face wrinkles; (8) coarse skin; (9) softness of skin; (10) elasticity of skin; (11) glossy skin; (12) overall complexion of skin. This is a non-validated questionnaire. Participants were asked to mark vertically on the SKN-VAS line for each question. The Anti-Aging Common Questionnaire (AAQOL) was also collected and consisted of 32 question items of physical symptoms, 21 questions concerning mental symptoms, and lifestyle-related questions, including sleep, drinking, smoking, and exercise habits. Both physical and mental symptoms were collected on a five-point Likert scale. They are grouped under the following perceptions.

VDT-related symptoms: tired eyes, blurry eyes, eye pain, stiff shoulders, muscle pain/strain, lethargy, and headache;

Fatigue-related symptoms: overweight, lethargy, no feeling of good health, appetite loss, early satiety, and epigastralgia;

Persistent neurological symptoms: palpitation, thirst, headache, dizziness, tinnitus, lumbago, and arthralgia;

Depression-related symptoms: irritability, short temper, reluctance to talk, depression, feeling of uselessness, shallow sleep, and difficulty falling asleep;

Loss of self-confidence: loss of motivation, no feeling of happiness, nothing to look forward to, daily life is not enjoyable, loss of confidence, pessimism, and anxious before sleeping;

Anxiety-related symptoms: lapse of memory, inability to concentrate, inability to solve problems, inability to decide, a sense of tension, anxiety without reasons, and a vague feeling of fear;

Autonomic nerve-related symptoms: dizziness, tinnitus, oedema, sweating, frequent urination, hot flush, and cold sensation;

Fragile constitution: weight loss, skin problems, weak chest, coughing and sputum, diarrhea, constipation, and cold sensation.

### 4.9. Statistical Analysis

Data are reported as mean ± SD unless otherwise specified. Data were analyzed via a three (3)-way [Smoking (Smokers vs. Non-smokers) × Condition (Solarplast^®^ vs. Placebo) × Time (Pre vs. Post)] analysis of variance (ANOVA) for metabolic, inflammatory, and anti-oxidant readouts. The normality of model residuals was assessed by Shapiro–Wilk tests along with visual inspection of the quantile-quantile plots. For data collected from the questionnaires, we assessed the degree of change between the beginning and the end of the study following 45 days of Solarplast^®^ administration A negative score suggests a decrease in the respective readout. These data were analyzed using a two-way ANOVA [Smoking (Smokers vs. Non-smokers) × Condition (Solarplast^®^ vs. Placebo)] to reduce violations due to interval data. In the event of a significant effect of treatment or smoking or an interaction effect, post-hoc comparisons were made with a Tukey adjustment. Significance was set a priori at an alpha level of *p* ≤ 0.05. The statistical analyses were performed using SPSS Version 27.

## 5. Conclusions

This is the first randomized, placebo-controlled trial conducted on the enzymatically enhanced spinach supplement, Solarplast^®^. Herein, we characterized the antioxidant potential of Solarplast^®^ as determined by total phenolic compounds, ORAC assessment (Oxygen Radical Absorbance Capacity), DPPH assessment (2,2-diphenyl-1-picrylhydrazyl), FRAP analysis (Ferric Reducing Antioxidant Power), and flavonoid analysis. We further investigated the impact of this product on serum antioxidant and inflammatory cytokine levels using a cohort of daily smokers within the study design as a proxy of oxidative stress exposure. Solarplast^®^ significantly attenuated smoking-induced changes in antioxidant biomarkers including glutathione and the ROS/RNS ratio. Similarly, Solarplast^®^ significantly attenuated smoking-induced changes in the inflammatory cytokines IL-6 and TNFα and also decreased the proinflammatory cytokine TNFα in the non-smoking group. Using a self-reported skin visual analog assessment, Solarplast^®^ improved participants’ perceptions of glossy skin and elasticity of skin. In the anti-ageing QOL common questionnaire, 45-day Solarplast^®^ supplementation reduced self-reported physical symptoms including blurry and tired eyes, sweating, and incidence of coughing and sputum production as well as reduced skin complaints. In the mental assessment of the anti-ageing QOL common questionnaire, Solarplast^®^ reduced perceived irritability, easiness to anger, reluctance to talk to strangers, loss of motivation, loss of confidence, inability to sleep because of worries, nothing to look forward to, perceived sense of tension, feelings of anxiety for no specific reason, and feelings of depression after 45 days of daily supplementation.

Solarplast^®^ was well-tolerated with no participants reporting adverse effects associated with the supplement during the 45-day daily consumption period. Further, clinical, hematological biomarkers related to health were examined, with no abnormal results reported due to the 45-day supplementation period. Mean values for the comprehensive metabolic panel were all within normal clinical limits pre- and post-supplementation for both non-smokers and smokers. It can be concluded that Solarplast^®^ is safe for consumption by healthy adults.

These findings suggest that Solarplast^®^ may play a beneficial physiological role that facilitates improvements in mood, skin, and physical readouts and appears to positively impact subjective skin health and quality of life metrics. While these physiological benefits are hard to study, our initial positive results with Solarplast^®^ supplementation suggest they should be further examined.

## Figures and Tables

**Table 1:** The antioxidant characterization results according to different spectrophotometric methodologies (FRAP, DPPH, ORAC, and Total Polyphenols), complementing previous published results [11].

Antioxidant Evaluation	Solarplast^®^
FRAP (mM TE/g, dry basis)	115.8 ± 7.9
DPPH test (U TE/g, dry basis)	742.8 ± 32.7
ORAC (mM TE/g, dry basis)	27.0 ± 6.8
TPC (GAE, as % dry basis)	3.3 ± 1.4

TE (Trolox equivalents), U (µmol/L/min), GAE (gallic acid equivalents).

**Table 2:** The tentative identification of phenolic components of Solarplast^®^.

Retention Time (min)	Molecular Weight	MS [M-H]^−^ *m*/*z*	MS^2^ [M-H]^−^ *m*/*z*	Compounds
Phenolic acids				
5.1	180	179.0568	161.0476	Caffeic acid
5.1	198	197.8081		Syringic acid
Flavonoids				
44.9	788	787.1952	332.0499	Patuletin *
46.3	935	934.2396	331.0445	Patuletin *
46.7	802	801.2115	346.0633	Spinacetin ^¥^
47.0	657	656.1593	331.0555	Patuletin *
47.1	318	317.0277		Myricetin
49.5	671	670.1731	346.0642	Spinacetin ^¥^
49.6	302	301.0332		Quercetin
51.3	448	448.1897		Luteolin-7-O-glucoside
53.7	610	609.0482		Quercetin-3-O-rutinoside
60.2	432	431.2255		Apigenin-7-O-glucoside
65.0	286	285.2692	174.9548	Luteolin
65.8	464	463.2931		Quercetin-3-O-glucoside
67.2	578	577.3579		Apigenin-7-O-rutinoside
67.3	594	593.3925	283.2637	Luteolin-7-O-rutinoside
67.8	272	271.2286		Naringenin
68.0	270	269.2468		Apigenin
68.1	286	285.2698		Kaempferol

* Patuletin species: at 44.9 min (Patuletin-3-O-*β*-D-glucopyranosyl-(1→6)-β-D-apiofuranosyl-(1→2)]-β-D-glucopyranoside), at 46.3 min (Patuletin-3-O-β-D-(2″-p-coumaroylglucopyranosyl-(1→6)-[β-D-apiofuranosyl-(1→2)]-β-D-glucopyranoside, at 47 min (Patuletin-3-O-β-D-glucopyranosyl-(1→6)-β-D-glucopyranoside). ^¥^ Spinacetin species: at 46.7 min (Spinacetin-3-O-β-D-glucopyranosyl-(1→6)-[β-D-apiofuranosyl-(1→2)]-β-Dglucopyranoside), at 49.5 min (Spinacetin-3-O-β-D-glucopyranosyl-(1→6)-β-D-glucopyranoside).

**Table 3:** Smoker and non-smoker participant group characteristics (mean ± SD).

	Solarplast^®^ Non-Smoker	Placebo Non-Smoker	Solarplast^®^ Smoker	Placebo Smoker
Age (yrs)	25.7 ± 9.7	27.2 ± 13.4	28.1 ± 13.9	31.6 ± 13.4
Males	*n* = 13	*n* = 12	*n* = 4	*n* = 4
Females	*n* = 20	*n* = 23	*n* = 4	*n* = 4
Height (cm)	168.0 ± 9.3	165.5 ± 8.3	167.7 ± 16.7	167.3 ± 9.5
Body Mass (kg)	71.7 ± 17.4	67.6 ± 13.2	73.4 ± 18.6	76.4 ± 16.0
Body Fat (%)	27.4 ± 4.6	26.1 ± 5.4	26.2 ± 6.5	29.3 ± 6.5

**Table 4:** There were no significant effects of 45-day Solarplast^®^ treatment, smoking, or any interaction with respect to time in any of the metabolic readouts (mean ± SD).

	Placebo Non-Smoker		Placebo Smoker		Solarplast^®^ Non-Smoker		Solarplast^®^ Non-Smoker	
	Pre	Post	Pre	Post	Pre	Post	Pre	Post
Glucose (mg/dL)	87.94 ± 7.16	88.80 ± 6.50	92.88 ± 5.49	91.63 ± 6.48	90.34 ± 7.55	90.76 ± 7.22	90.34 ± 7.55	90.76 ± 7.22
BUN (mg/dL)	12.60 ± 3.98	11.97 ± 3.65	12.13 ± 3.72	10.75 ± 2.38	12.09 ± 3.05	12.36 ± 3.89	12.09 ± 3.05	12.36 ± 3.89
Creatinine (mg/dL)	0.85 ± 0.15	0.84 ± 0.15	0.85 ± 0.19	0.84 ± 0.18	0.87 ± 0.19	0.91 ± 0.19	0.87 ± 0.19	0.91 ± 0.19
EGFR (mL/min/1.73)	105.76 ± 15.3	106.61 ± 13.1	105.0 ± 14.2	104.63 ± 9.3	102.77 ± 18.1	101.25 ± 16.7	102.77 ± 18.1	101.25 ± 16.7
BUN/Creatinin	14.89 ± 4.23	14.37 ± 4.25	14.38 ± 4.21	12.88 ± 3.14	13.94 ± 2.93	13.70 ± 3.40	13.94 ± 2.93	13.70 ± 3.40
Sodium (mmol/L)	139.60 ± 1.79	139.34 ± 2.14	140.50 ± 1.20	141.13 ± 1.73	140.21 ± 2.15	140.12 ± 2.55	140.21 ± 2.15	140.12 ± 2.55
Potassium (mmol/L)	4.64 ± 0.52	4.56 ± 0.59	4.23 ± 0.40	4.28 ± 0.20	4.39 ± 0.33	4.49 ± 0.44	4.39 ± 0.33	4.49 ± 0.44
Chloride (mmol/L)	102.00 ± 2.10	102.31 ± 2.19	103.13 ± 2.03	102.38 ± 1.51	102.64 ± 2.03	102.33 ± 2.04	102.64 ± 2.03	102.33 ± 2.04
Carbon Dioxide (mmol/L)	22.31 ± 2.26	22.51 ± 2.42	22.75 ± 1.67	20.75 ± 2.38	22.82 ± 1.89	22.03 ± 1.78	22.82 ± 1.89	22.03 ± 1.78
Calcium (mg/dL)	9.57 ± 0.34	9.47 ± 0.33	9.56 ± 0.30	9.48 ± 0.36	9.52 ± 0.39	9.54 ± 0.33	9.52 ± 0.39	9.54 ± 0.33
Total Protein (g/dL)	7.31 ± 0.42	7.17 ± 0.36	7.34 ± 0.54	7.33 ± 0.54	7.29 ± 0.39	7.33 ± 0.36	7.29 ± 0.39	7.33 ± 0.36
Albumin (g/dL)	4.75 ± 0.32	4.70 ± 0.29	4.69 ± 0.32	4.64 ± 0.37	4.62 ± 0.29	4.68 ± 0.25	4.62 ± 0.29	4.68 ± 0.25
Globulin (g/dL)	2.56 ± 0.30	2.47 ± 0.30	2.65 ± 0.48	2.56 ± 0.47	2.66 ± 0.31	2.65 ± 0.34	2.66 ± 0.31	2.65 ± 0.34
A/G Ratio	1.88 ± 0.25	1.94 ± 0.32	1.84 ± 0.41	1.88 ± 0.33	1.76 ± 0.25	1.79 ± 0.27	1.76 ± 0.25	1.79 ± 0.27
Bilirubin (mg/dL)	0.57 ± 0.32	0.56 ± 0.33	0.53 ± 0.33	0.44 ± 0.27	0.60 ± 0.45	0.59 ± 0.8	0.60 ± 0.45	0.59 ± 0.8
Alkaline Phosphatase (IU/L)	69.83 ± 24.41	68.09 ± 22.08	71.25 ± 20.30	75.50 ± 20.63	64.06 ± 15.92	67.58 ± 16.08	64.06 ± 15.92	67.58 ± 16.08
AST (IU/L)	27.29 ± 15.80	23.17 ± 8.17	20.50 ± 10.21	23.25 ± 7.59	21.06 ± 7.52	22.64 ± 7.17	21.06 ± 7.52	22.64 ± 7.17
ALT (IU/L)	19.89 ± 9.74	19.89 ± 14.65	23.13 ± 14.54	25.63 ± 15.53	19.67 ± 12.20	19.21 ± 9.34	19.67 ± 12.20	19.21 ± 9.34

**Table 5:** Change from baseline in skin visual analog results for smokers and non-smokers following 45 days of Solarplast^®^ or placebo supplementation.

	Placebo Non-Smoker	Placebo Smoker	Solarplast^®^ Non-Smoker	Solarplast^®^ Smoker
Dryness	−0.09 ± 0.18	−0.25 ± 0.31	−0.35 ± 0.24	−0.38 ± 0.47
Flushing	−0.11 ± 0.16	−0.38 ± 0.38	−0.56 ± 0.20	−0.44 ± 0.29
Inconsistency of skin with make-up	0.20 ± 0.13	0.13 ± 0.13	−0.12 ± 0.14	0.25 ± 0.25
Itching of skin	0.03 ± 0.17	−0.25 ± 0.31	−0.32 ± 0.24	−0.13 ± 0.40
Eczema	−0.01 ± 0.04	0.25 ± 0.16	−0.27 ± 0.15	0.00 ± 0.00
Body wrinkles	0.06 ± 0.07	0.13 ± 0.13	−0.09 ± 0.09	−0.13 ± 0.13
Face wrinkles	−0.09 ± 0.06	0.00 ± 0.27	−0.12 ± 0.08	−0.34 ± 0.25
Coarse skin	0.04 ± 0.10	−0.13 ± 0.13	−0.15 ± 0.16	0.00 ± 0.00
Softness of skin	−0.21 ± 0.22	−0.63 ± 0.50	0.29 ± 0.17	−0.13 ± 0.69
Elasticity of skin ^A^	−0.17 ± 0.21	−0.88 ± 0.58	0.41 ± 0.25	0.38 ± 0.71
Glossy skin ^A^	−0.04 ± 0.21	−0.63 ± 0.38	0.24 ± 0.32	0.88 ± 0.67
Complexion of skin	0.14 ± 0.19	−0.63 ± 0.65	0.35 ± 0.15	0.00 ± 0.53

^A^ represents an overall effect of treatment (*p* < 0.05). All data are mean ± SEM.

**Table 6:** Changes in the anti-ageing quality of life common questionnaire physical symptoms in non-smokers and smokers who consumed Solarplast^®^ or placebo for 45 days (mean ± SEM).

	Placebo Non-Smoker	Placebo Smoker	Solarplast^®^ Non-Smoker	Solarplast^®^ Smoker
Tired Eyes ^A^	−0.06 ± 0.14	0.38 ± 0.46	−0.30 ± 0.15	−0.50 ± 0.27
Blurry Eyes ^A^	0.11 ± 0.11	−0.25 ± 0.16	−0.30 ± 0.13	−0.75 ± 0.25
Eye Pain	−0.11 ± 0.09	−0.13 ± 0.13	−0.15 ± 0.08	−0.13 ± 0.13
Stiff Shoulders ^B^	0.11 ± 0.14	−0.50 ± 0.27	−0.27 ± 0.15	−0.75 ± 0.31
Muscular Pain	0.14 ± 0.13	−0.13 ± 0.30	−0.27 ± 0.12	−0.50 ± 0.19
Headache	−0.40 ± 0.17	0.13 ± 0.30	−0.30 ± 0.15	−0.38 ± 0.26
Lethargy	−0.11 ± 0.14	−0.25 ± 0.31	−0.18 ± 0.13	−0.38 ± 0.38
Perceived Weight Gain	0.03 ± 0.10	−0.13 ± 0.23	−0.21 ± 0.17	−0.75 ± 0.45
No Feelings of Good Health	0.14 ± 0.11	0.25 ± 0.25	0.03 ± 0.15	−0.25 ± 0.25
Perceived Weight Loss	0.00 ± 0.09	0.00 ± 0.00	−0.06 ± 0.07	0.00 ± 0.19
Early Satiety	−0.03 ± 0.13	0.13 ± 0.13	−0.21 ± 0.11	0.00 ± 0.00
Epigastralgia	0.00 ± 0.06	−0.13 ± 0.13	−0.06 ± 0.04	0.00 ± 0.00
Palpitations	0.00 ± 0.00	0.00 ± 0.00	−0.03 ± 0.03	−0.13 ± 0.13
Thirst ^B^	−0.03 ± 0.12	−0.75 ± 0.37	−0.27 ± 0.15	−0.75 ± 0.49
Dizziness	0.03 ± 0.09	0.25 ± 0.16	−0.18 ± 0.09	−0.13 ± 0.13
Tinnitus	0.06 ± 0.07	0.00 ± 0.00	−0.06 ± 0.04	0.00 ± 0.00
Lumbago	−0.14 ± 0.12	0.25 ± 0.41	−0.30 ± 0.12	0.00 ± 0.00
Arthralgia	−0.03 ± 0.05	−0.25 ± 0.25	−0.09 ± 0.05	0.00 ± 0.00
Oedema	0.03 ± 0.03	0.00 ± 0.00	−0.03 ± 0.03	0.00 ± 0.00
Sweat ^A^	−0.09 ± 0.10	0.13 ± 0.35	−0.24 ± 0.13	−0.50 ± 0.19
Frequent Urination	−0.06 ± 0.16	0.00 ± 0.33	−0.15 ± 0.09	−0.50 ± 0.27
Hot Flushes	0.09 ± 0.10	0.13 ± 0.13	−0.09 ± 0.08	0.00 ± 0.00
Cold Intolerance	−0.06 ± 0.11	0.00 ± 0.19	−0.18 ± 0.13	−0.38 ± 0.18
Prone to Illness	0.00 ± 0.15	0.00 ± 0.00	−0.09 ± 0.08	0.00 ± 0.00
Cough and/or Sputum ^A^	0.14 ± 0.15	0.00 ± 0.00	0.06 ± 0.04	−0.88 ± 0.40 *′
Diarrhea	0.06 ± 0.09	0.00 ± 0.00	−0.03 ± 0.05	0.00 ± 0.00
Constipation	−0.09 ± 0.09	−0.13 ± 0.13	−0.03 ± 0.09	−0.13 ± 0.13
Skin Problems ^A^	0.06 ± 0.10	0.00 ± 0.27	−0.64 ± 0.19 ^†^	−0.38 ± 0.32

^†^ represents a significant post-hoc difference between the non-smoking placebo and non-smoking Solarplast^®^-treated groups (*p* < 0.05). *′ represents a significant post-hoc difference between the smoking Solarplast^®^-treated groups (*p* < 0.05) against both the non-smoking Solarplast^®^-treated group and smoking, placebo-treated group (*p* < 0.05). ^A^ represents an overall effect of treatment, while ^B^ represents an overall effect of smoking as determined by two-way ANOVA.

**Table 7:** Changes in the anti-ageing quality of life common questionnaire for mental symptoms in non-smokers and smokers who consumed Solarplast^®^ or Placebo for 45 days (mean ± SEM).

	Placebo Non-Smoker	Placebo Smoker	Solarplast^®^ Non-Smoker	Solarplast^®^ Smoker
Irritability ^A^	0.00 ± 0.14	−0.38 ± 0.32	−0.45 ± 0.14	−0.88 ± 0.30
Easily angered ^A^	−0.06 ± 0.09	0.00 ± 0.00	−0.42 ± 0.12	−0.88 ± 0.30 ^†^
Reluctance to talk with others ^A^	−0.14 ± 0.10	0.38 ± 0.42	−0.12 ± 0.13	−0.50 ± 0.27
Feeling depressed ^A^	0.03 ± 0.09	0.38 ± 0.26	−0.15 ± 0.08	−1.00 ± 0.46 *′
Feeling of uselessness ^B^	−0.17 ± 0.24	−1.13 ± 0.48	−0.15 ± 0.33	−1.00 ± 0.42
Shallow sleep	−0.14 ± 0.18	0.25 ± 0.37	−0.15 ± 0.19	−0.25 ± 0.59
Difficulty falling asleep	−0.11 ± 0.17	−0.63 ± 0.38	−0.27 ± 0.21	−0.38 ± 0.26
Loss of motivation ^A^	−0.09 ± 0.10	−0.13 ± 0.30	0.00 ± 0.18	−0.88 ± 0.23 *
No feeling of happiness ^A^	−0.06 ± 0.09	0.13 ± 0.23	−0.09 ± 0.11	−0.50 ± 0.27
Nothing to look forward to ^A^	0.03 ± 0.03	0.25 ± 0.16	0.03 ± 0.05	−0.38 ± 0.26 *
Daily life not enjoyable	0.03 ± 0.09	0.13 ± 0.13	0.06 ± 0.06	−0.25 ± 0.25
Loss of confidence ^A^	−0.09 ± 0.10	0.00 ± 0.00	−0.39 ± 0.14	−0.50 ± 0.27
Pessimism	−0.14 ± 0.10	−0.13 ± 0.13	−0.03 ± 0.08	−0.63 ± 0.32
Inability to sleep because of worries	0.03 ± 0.11	1.00 ± 0.50 ^†^′	−0.39 ± 0.19	−0.38 ± 0.18
Lapse in memory	−0.06 ± 0.12	0.00 ± 0.27	−0.15 ± 0.12	−0.63 ± 0.50
Inability to concentrate	−0.20 ± 0.15	−0.38 ± 0.26	−0.33 ± 0.15	−0.50 ± 0.19
Inability to solve problems	0.00 ± 0.12	0.13 ± 0.13	−0.18 ± 0.13	0.13 ± 0.23
Inability to make judgement	−0.20 ± 0.11	−0.25 ± 0.16	0.00 ± 0.11	0.00 ± 0.19
Sense of tension ^A^	−0.20 ± 0.11	0.25 ± 0.41	−0.42 ± 0.16	−0.63 ± 0.38
Vague feeling of fear	−0.06 ± 0.11	−0.26 ± 0.12	−0.13 ± 0.13	0.00 ± 0.00
Feeling anxious for no reason ^A^	−0.11 ± 0.12	0.00 ± 0.53	−0.64 ± 0.19	−0.63 ± 0.26

^†^ represents a significant post-hoc difference between the smoking placebo and smoking Solarplast^®^-treated groups (*p* < 0.05). ^†^′ represents a significant post-hoc difference between the smoking placebo and both the smoking Solarplast^®^-treated groups and non-smoking placebo treated groups (*p* < 0.05). *′ represents a significant post-hoc difference between the smoking Solarplast^®^-treated groups (*p* < 0.05) against both the non-smoking Solarplast^®^-treated group and smoking, placebo-treated group (*p* < 0.05). * represents a significant post-hoc difference between the smoking Solarplast^®^-treated group and the non-smoking Solarplast^®^-treated group (*p* < 0.05). ^A^ represents an overall effect of treatment, while ^B^ represents an overall effect of smoking as determined by two-way ANOVA.

## Data Availability

Results of all analyses are included in this published article. The datasets generated and/or analyzed during the current study are available from the corresponding author on reasonable request.
